# Sandwich Panels Subjected to Point Loads: Design Approach Using Effective Widths in Elastic Range

**DOI:** 10.3390/ma18122910

**Published:** 2025-06-19

**Authors:** Niklas Ardelmann, Bernd Naujoks

**Affiliations:** Department of Steel- and Composite-Structures, University of Wuppertal, Pauluskirchstraße 11, 42285 Wuppertal, Germany

**Keywords:** sandwich panels, point loads, effective widths, cyclic loading, experimental investigations, numerical studies, parametric studies

## Abstract

Sandwich panels have established themselves as self-supporting and isolating construction elements for room closures in hall construction. As a result of subsequently installed photovoltaic (PV) systems or cladding, sandwich panels are subjected to point loads at the connection points to the substructure of additional systems. In the case of pressure-suction changes from wind, a cyclical local load also occurs. Therefore, for sandwich panels—which are designed and dimensioned for uniform surface loads (dead weight, wind, snow, temperature constraints)—the question must be answered as to how this local load introduction affects the load-bearing behaviour and stress distribution in the sandwich panel. To quantify any stress concentrations across the width of the panel, the method of effective widths is used here, based on stress distributions in the elastic range determined through component tests and numerical models. The results of these test series, along with the resulting design concept based on effective widths in the elastic range, as well as the failure under the ultimate load condition, are documented in this paper.

## 1. Introduction

### 1.1. Motivation

**Figure 1:**
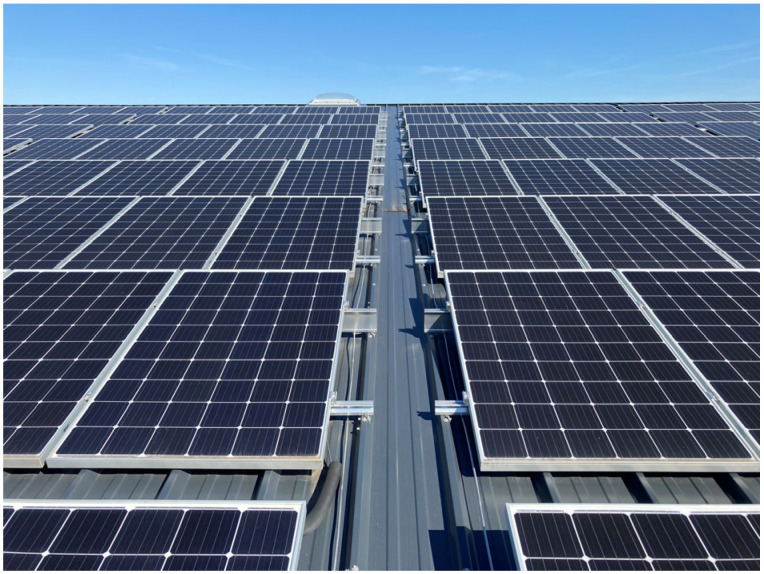
PV system on sandwich roof (Source: Ingenieurbüro Dr. Zapfe GmbH).

Point loads on sandwich panels commonly occur due to the installation of additional photovoltaic (PV) systems on roofs (see Figure 1).

The uniform loads—snow, wind, and self-weight—of the PV system are then introduced and concentrated at the connection points between the sandwich panel and the PV rails. For tilted PV modules, significant pulling forces occur; pressure-suction changes are also important for the design from a durability point of view.

**Figure 2:**
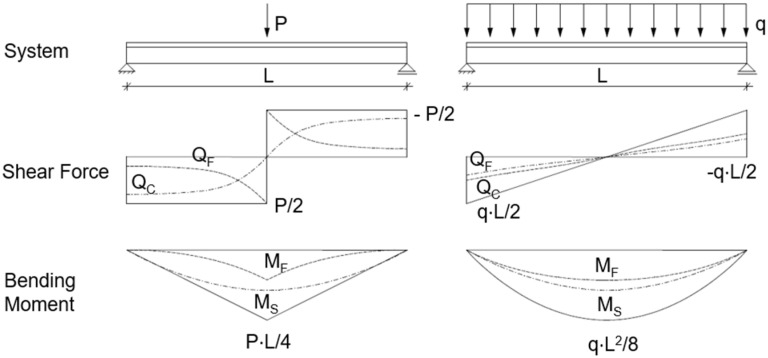
Qualitative internal forces from transverse line toad and uniform load on one-span 2D sandwich beam with profiled face.

The point load can be idealized as a transversal line load that is not acting across the full width. For a transversal line load acting across the full width, the stress resultants are already derived, for example by Stamm/Witte [1]. The fundamentals of the sandwich theory have also been presented by Plantema [2], Allen [3], and Davies [4]. For sandwich panels with one profiled face, the internal forces differ significantly from those of a longitudinal line load acting across the full width, as qualitatively shown in Figure 2.

It can be seen that, in the area of the point load application, the entire shear force is transferred via the profiled face (Q_F_). With increasing distance from the load application, the load-bearing components shift toward the core (Q_C_). As a result, the face bending moment (M_F_) integrated from shear force Q_F_ also contributes less to the moment transfer at a distance from the point load. Thus, the sandwich principle (M_S_) takes over the majority of the load bearing.

These changes in load-bearing components are not observed with the uniform line load, where both load-bearing principles contribute equally over the entire span.

We can already conclude that even a point load, which could activate the full width of a panel, has far more sophisticated courses of internal forces than a uniformly distributed load.

### 1.2. State of Research and Design

All current design formats and component resistances are based on the assumption of uniform cross-sectional activation of the sandwich panel in the width direction. The interaction of vertical compression and wrinkling at the mid-support, which activates the full panel width almost equally, has already been investigated by Nelke [5] and Engel [6]. As the premise of full cross-section activation is no longer fulfilled for point loads, verification of the load-bearing capacity under point loads is currently only possible via approvals in individual cases or type approvals. This requires time- and cost-consuming ultimate-load tests for the specific sandwich panel installed on the roof, which are usually difficult to provide for older existing roofs. As part of the new generation of Eurocode 3 standards for steel structures, a design part specifically for sandwich panels is being introduced for the first time as Part 7 [7]. This also includes consideration of point loads using effective widths based on trapezoidal sheets (DIN 18807-3 [8]) for bending and shear. However, the formulae for effective widths must be adapted using component-specific coefficients/correction factors for bending and shear, which the manufacturer must specify in the product approval. The first approvals already contain these coefficients. There, the coefficients always result in the maximum value of 1.0, so that the effective widths of the trapezoidal sheets can be adopted without adjustment. It should be noted that the coefficients are based solely on the ultimate load state, which does not provide any information about the actual cross-sectional activation in the elastic range, which is essential for the superposition with other uniform load cases acting simultaneously.

Furthermore, excentric point loads cause torsion in the sandwich panel. Torsion is not explicitly discussed in this research paper but has been thematized in [9]. The load-bearing behaviour of construction sandwich panels subjected to torsion has been investigated in depth by Pradhan [10,11], Pozorski [12,13], and others.

### 1.3. Scope of the Research Project

The aim of the investigations at the Institute of Structural Engineering at the University of Wuppertal was therefore to initially quantify the normal stress distribution in the loading axis and the longitudinal spreading in the faces in the elastic range by applying a dense arrangement of strain gauges (SGs) in component tests on a two-span girder. Additionally, the load-bearing behaviour in the overcritical range was investigated through ultimate load tests. In a separate series of tests, a cyclic punctual compression–tension load was also applied in order to determine any load-reducing effects. The elastic shear stress distribution in the profiled face and core was investigated in validated numerical models based on the measured values. Both wall (L) and roof panels (T) with core thicknesses (d) of 140 mm and 60 and 140 mm, respectively, as well as the two core materials polyisocyanurate (PIR) and mineral wool (MW), were considered in the investigations.

With knowledge of the stress distributions, a purely calculation-based design format was then developed, based on the existing component resistances applicable for uniform loads and an increase in the actions over effective widths at specific stress levels for each stress resultant. This approach is intended to enable cross-manufacturer transferability.

## 2. Experimental Investigations

### 2.1. Test Setup

**Figure 3:**
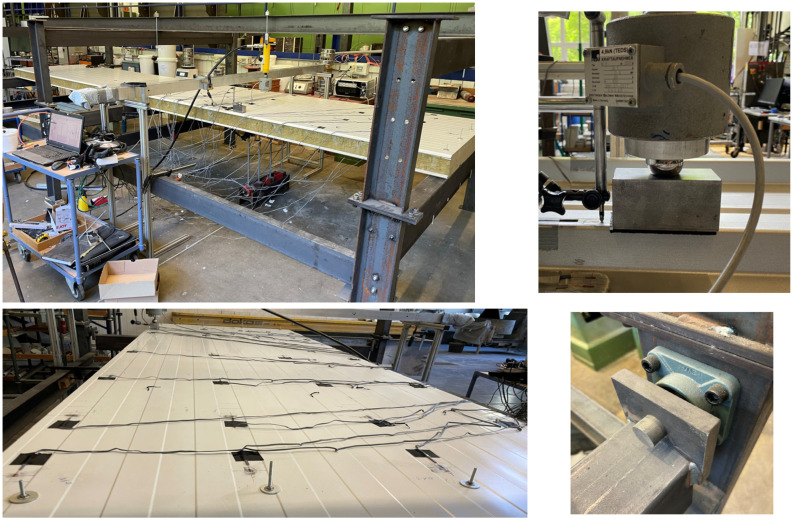
Test setup.

The component tests were all carried out on a two-span girder (2 × 3 m) in order to gain insights into cross-section activation at the mid-support, which is often relevant for design in the SLS (Figure 3).

**Figure 4:**
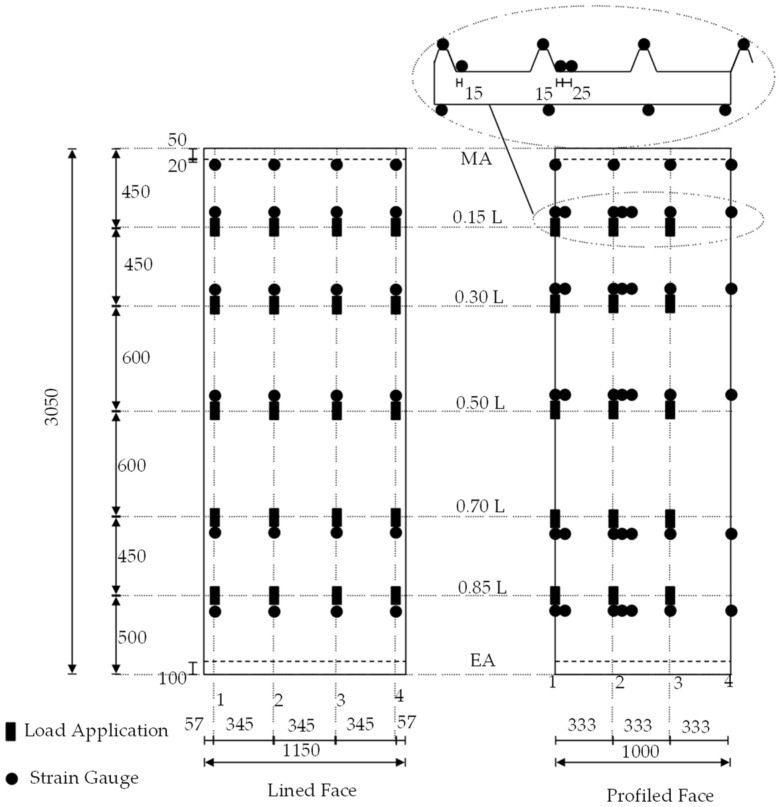
Load positions and strain gauge positions.

In order to ensure hinged support, the sandwich panels were attached to 100 mm wide rectangular hollow profile purlins, which were connected to the frame with radial bearings. With a width of 2.500 mm, the test stand was sufficiently wide to also examine two sandwich panels coupled next to each other. The point load was applied via a manually operated hydraulic cylinder, to which a force measuring cell was connected. The cylinder was connected to a load traverse (IPE 140) via threaded rods. This design allowed the sandwich panel to be loaded at any desired position. In the longitudinal direction, the load positions 0.15, 0.30, 0.50, 0.70, and 0.85 L (measured from the mid-support) were considered; in the transverse direction, each core-supported trapezoidal rib was loaded. This resulted in a total of 3 × 5 load positions for the roof elements and 4 × 5 load positions for the wall elements (Figure 4). The load was applied to the sandwich panel via a steel block, with a 50 × 100 mm contact area and an elastomer layer. A steel ball was interposed between the steel block and load cell in order to allow for free rotation.

The component tests focused on recording the normal stress distribution in the faces. For this purpose, a total of 63 strain gauges—which were always orientated in the longitudinal direction of the panel—were used in the loaded span for each roof element in the load axes and at the mid-support. For the wall elements, 48 strain gauges were used due to the non-existent bending components of the lined face sheet. The load positions and the strain gauge applications are shown in the following figure, separated for the flat/lined and profiled faces.

The maximum load for the tests in the elastic range was set to 1.2 kN for all sandwich panel configurations. The deformations were recorded only in the currently loaded axis, with four displacement transducers at the bottom and one at the load introduction.

### 2.2. Tests in the Static Elastic Range

#### 2.2.1. Stresses

**Figure 5:**
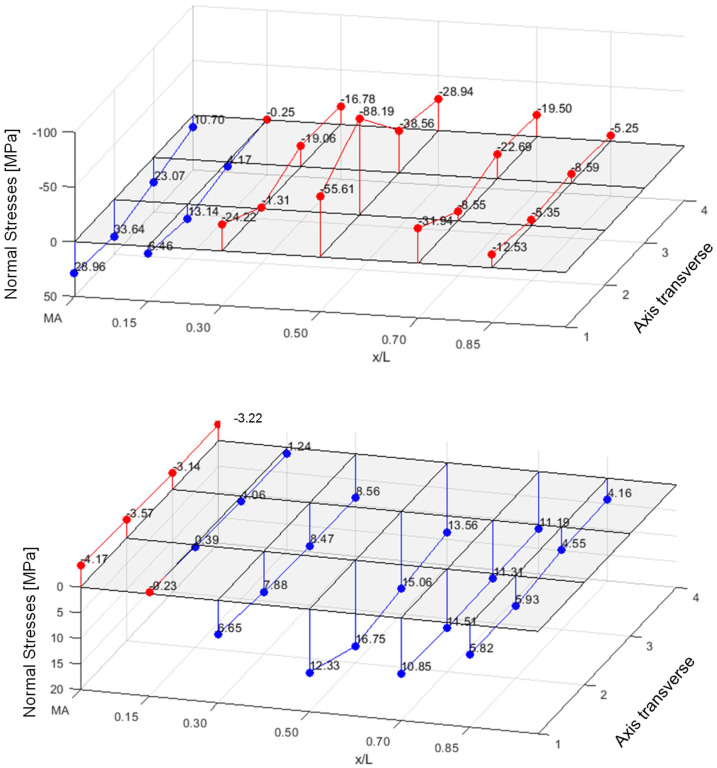
Normal stresses in upper flanges of ribs and lower face.

Figure 5 shows the normal stresses [MPa] (PIR roof panel with 60 mm core thickness, PIR-T-60) in the upper flanges of the ribs and in the lower face under a central load of 1.2 kN.

In the lower face, where only the normal force from the sandwich principle acts, there is already a good stress distribution across the width, even in the load axis. In the neighbouring axes to the point load, complete stress spreading is observed in the lower face.

The upper face, however, where the normal force from the sandwich principle and—due to the profiling—a bending moment of the face act, exhibits significant stress peaks in the load axis. The additional strain gauges applied to the base of the trapezoidal rib also detect significant bending tensile stresses from the bending moment in the rib.

Due to the decrease in the face bending moment along the longitudinal direction of the panel, as well as the stress propagation in the transverse direction, a significantly lower stress level is already observed in the neighbouring axes at a distance of 600 mm from the acting point load. Following the stresses in the loaded rib reveals a relieving effect from the point load in the neighbouring sections. This is due to a superposition of the global stress resultants of the sandwich panel with those of the rib, acting as an elastically bedded beam. This effect can be considered favourable for the frequently occurring case of several point loads within one span on a rib.

The stress pattern clearly shows that cross-section activation for bending needs to be divided into the relatively constant normal force and the concentrated bending moment in the profiled face.

When a roof panel is loaded at one point with an adjacent, connected roof panel (bolted together every 600 mm in the longitudinal direction), a relieving effect occurs, as the neighbouring element is also activated (elastic line support at the joint). When the load is applied to the overlapping rib itself, both panels are used almost equally to transfer the load. Therefore, analysing only one solitary panel is always a conservative approach. In any case, generalisations would not be possible due to the manufacturer-dependent joint geometry.

**Figure 6:**
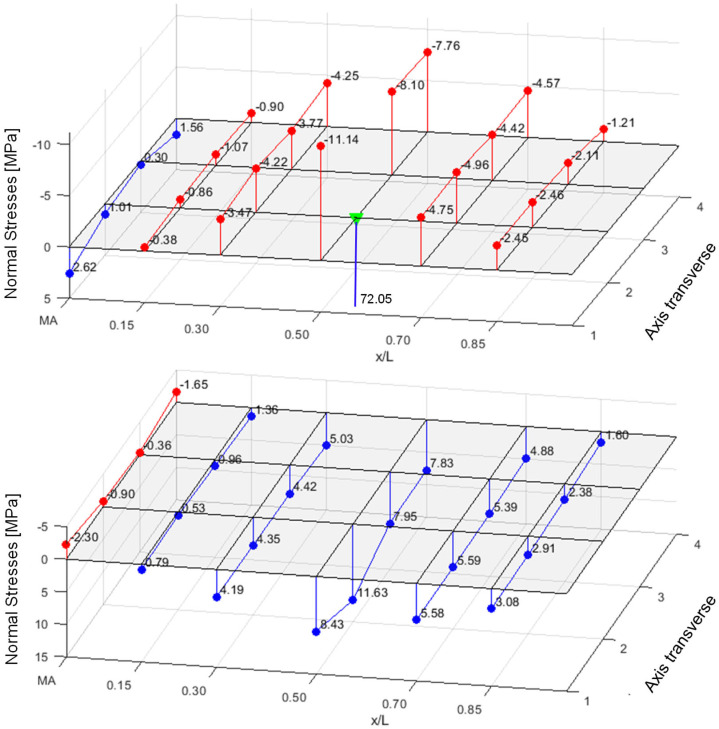
Normal stresses in upper and lower faces of a wall panel.

In the case of the wall panels, considerable membrane and bending stresses (up to 170 MPa tensile) can be observed in the flat face at 1.2 kN directly upon load introduction. The global stress level is low in comparison, as shown in Figure 6 (PIR-L-140).

#### 2.2.2. Effective Widths

Based on the normal stress distribution in the faces, effective widths were derived. The following figure illustrates the concept used in this context.

**Figure 7:**
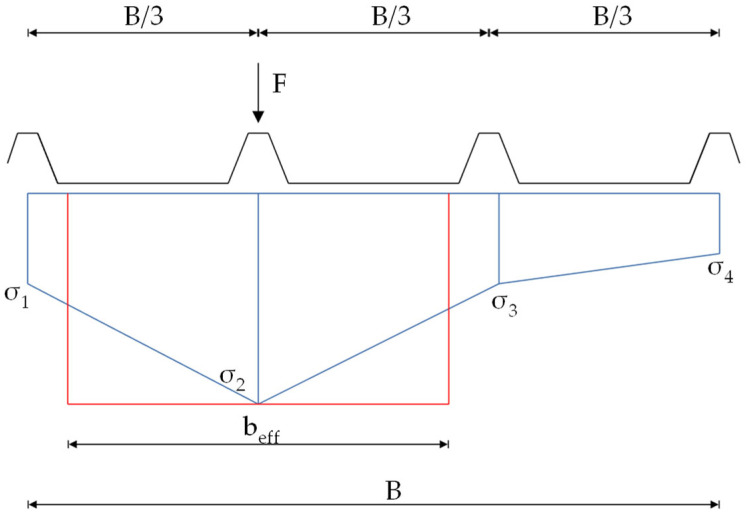
Concept of effective widths based on stresses.

Under punctual loading, a non-linear stress curve occurs in the direction of the width. This non-linear curve is approximated in this work by connecting the discrete stress points linearly with each other. The resulting trapezoidal stress areas, integrated over the total width B, are converted to an equivalent constant rectangular stress block, with the maximum stress amplitude over an effective width b_eff_ (Figure 7).

For the example depicted in Figure 7, b_eff_ would be calculated as follows:

(1) $$b_{eff}=\left(\frac{\sigma_{1}+\sigma_{2}}{2}\cdot \frac{B}{3}+\frac{\sigma_{2}+\sigma_{3}}{2}\cdot \frac{B}{3}+\frac{\sigma_{3}+\sigma_{4}}{2}\cdot \frac{B}{3}\right)/max\left(\sigma_{1-4}\right)$$

The reason for this approach is because of the different effective widths for the bending moment and the normal force, as seen in Figure 4 and Figure 5. To filter out which portion of the determined stresses results from the bending moment and which from the normal force, the following procedure has been used for the roof panels.

Since the lower lined face experiences only the normal force, the four stress points along the loaded axis on this face are used to calculate the effective width for the normal force. The upper profiled face of the roof elements undergoes bending and normal force loading. In the first step, the total normal stress measured in the upper flange of the rib is reduced by the normal stress from the corresponding stress point on the lower face, scaled by the area ratio of both faces. This yields the pure bending stress. Based on the resulting pure bending stresses, the effective width of the bending moment is then calculated. This procedure is essential for accurate analysis.

**Figure 8:**
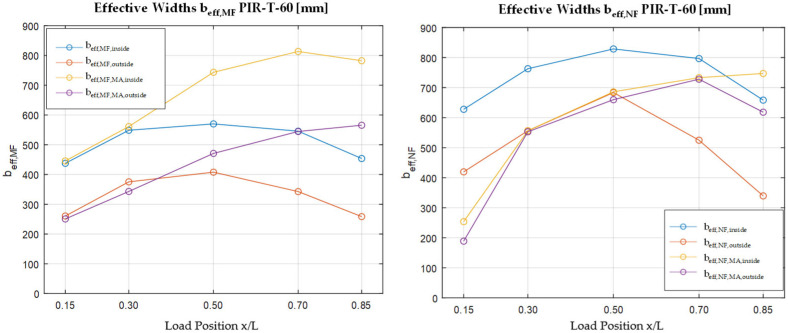
Resulting effective widths for bending moment and normal force.

Figure 8 shows the resulting effective widths for the bending moment and the normal force in the face. The courses are categorized based on whether a load is applied to an edge rib or an inner rib. It is later divided depending on whether the effective width at the mid-support or directly at the point load in the field is of interest. The position x represents the distance to the mid-support. Figure 7 shows the results for the PIR roof element with a core thickness of 60 mm.

Three results should be highlighted here. First, the effective widths of the normal force are larger than those of the bending moment, as already suggested by the normal stress patterns. Second, the effective widths are significantly smaller (up to 50%) when the load is applied on the edge. Third, the effective widths directly at the point load increase with growing distance to the supports; the effective width at the mid-support also increases.

The effective widths of the other sandwich configurations show almost equal courses and values. It can be concluded that the load position is the decisive factor for the effective widths.

#### 2.2.3. Deformations

**Figure 9:**
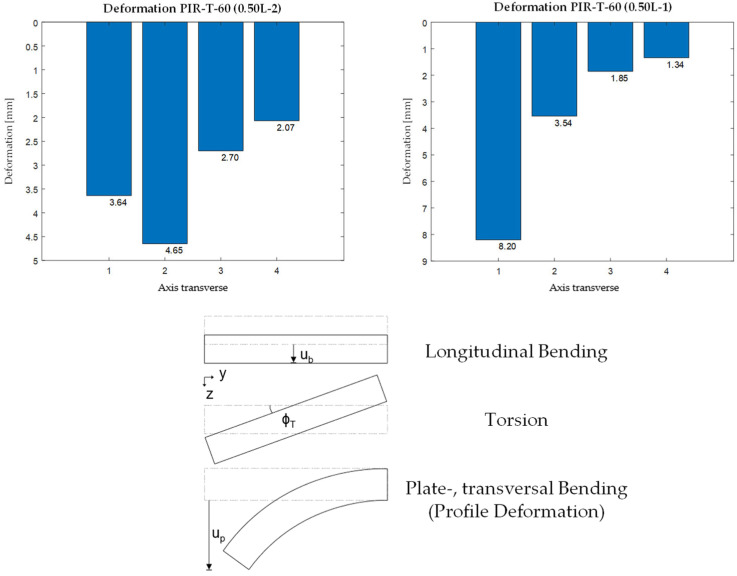
Deformation pattern and contributions.

The deformations were recorded only in the specifically loaded axis. The results show that cross-sectional uniformity is not maintained when loading is applied to an inner rib, and especially to an edge rib, as shown here using the 60 mm thick PIR roof panel (Figure 9).

The deformation pattern can be divided into three parts. In addition to the cross-sectionally consistent longitudinal deflection and torsional rotation, the core softness leads to a significant transverse bending or plate bending (Figure 9). This profile deformation has the effect that the determination of deformation under point loads on the one-dimensional shear–elastic beam does not satisfactorily reflect reality.

### 2.3. Ultimate Load Test

#### 2.3.1. Overview

After loading each panel in the elastic range with 1.2 kN at all load positions, a load-bearing test was carried out. The ultimate load tests were performed at three identical load positions for each roof panel type. First, the ultimate load was investigated along the midspan of an inner rib under compression and tension (four bolts on the web of the rib). The third test was conducted close to the mid-support (0.5 L) on an inner rib for compression to initiate shear failure, which did not occur in the later tests. The wall panels were not systematically tested for tension, as pre-tests showed only local failure due to bolt pull-out. The three compression tests until failure were executed at midspan (inside and edge) and at 0.15 L (inside).

**Figure 10:**
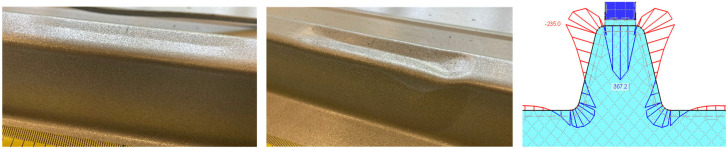
Local failure of rib from compression.

The failure of the panels can be divided into local and global failure. For the roof panels under compression, a crease appears in the upper flange of the rib from around 2.4 kN, regardless of the element configuration (Figure 10).

Through a numerical sub-model considering the radii of the steel face and a vertical load of 1.2 kN, the explanation for the crease can be derived. As shown in Figure 9 (right), the transversal bending behaves like a rigid frame, leading to a plastic hinge chain consistent with the observed local failure.

As the load increases, the folding increases before the web begins to deform plastically out of plane from around 4.0 kN (Figure 10 middle). It is worth mentioning that this is not a sudden stability failure of the rib accompanied by a drop in force.

For the wall panels, a local failure is not initially visible. The strain gauge applied directly at the load introduction indicates a relatively early surpassing of the panel’s yield strength at about 5 to 6 kN. This is especially true for the mineral wool type, where the upstanding fibers of the mineral wool are very likely to break due to vertical compression from the load introduction. The core does not provide any further support to the flat face, causing high local bending and membrane stresses.

However, the load-bearing capacity of the sandwich panel itself has not yet been elucidated in the aforementioned cases.

#### 2.3.2. Midspan and Mid-Support

**Figure 11:**
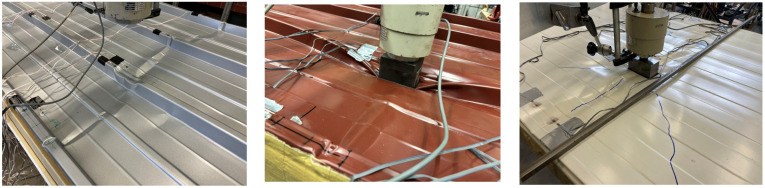
Global failure of panel from compression: PIR-T-60 9.4 kN (**left**); PIR-T-140 16.8 kN (**middle**); MW-L-140 9.0 kN (**right**).

**Figure 12:**
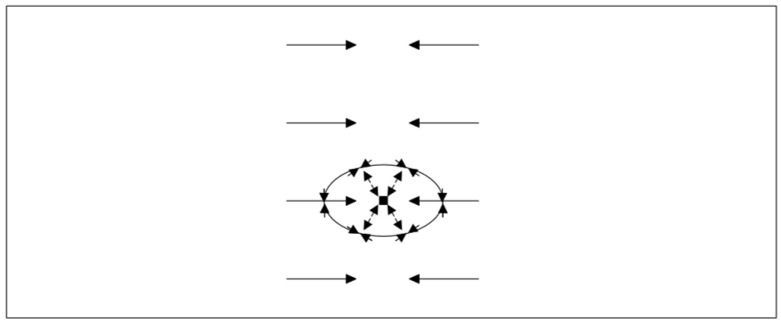
Local and global membrane effects on flat face.

The final failure occurs globally in the form of wrinkling of the neighbouring rib when a compression load is applied (Figure 11 left, middle). For wall panels, a continuous wrinkling crease in the load axis can be obtained (Figure 11 right). In addition, radial wrinkling creases result from the membrane effect (ref. to Figure 12).

In some cases, it is also possible to cause the other ribs to wrinkle one after the other with a further increase in load, creating a continuous crease in the profiled face. However, the loaded rib itself is already heavily plasticised at this stage and, in some cases, completely pressed into the core. The load-bearing loads thus significantly exceed the expected design loads at the connection points of a PV system, which are around 1.5 kN [14].

The radial wrinkling creases in the wall panels can be explained as follows: As a result of the indentation of the point load into the core, a trough forms. To maintain equilibrium in the upper face, a compression ring forms around the trough, which expands as the load and indentation increase. As a result of the point load being suspended in the face, the global compressive stresses are superimposed with tangential compressive stresses and radial tensile stresses from the membrane (see also Lübke [15]), as shown in the figure below.

**Figure 13:**
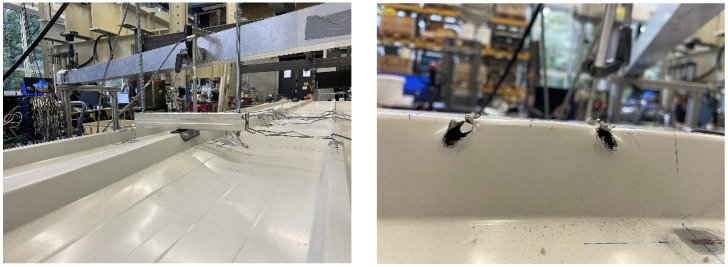
Global (**left**) and local failures (**right**) under tension (MW-T-140).

Under tension, failure occurs in the case of the connection with two pairs of screws on the web, as a result of delamination of the loaded rib (already well advanced in Figure 13).

The maximum load under tension is in the range of 4.4–5.9 kN for all investigated configurations and is therefore significantly lower than under compression. After an immediate drop in force due to delamination, the rib is pulled further away from the core as the cylinder continues to load it, before the bolt pull-out occurs due to significant hole elongation, marking the end of the test. For the frequently used connection with only one pair of bolts on the web, a purely local failure due to ovalising hole formation/peeling was observed at around 2.5 kN.

It is worth mentioning that the load-bearing behaviour of the sandwich panel under tension in the elastic range does not differ from that under compression. With the exception of the immediate load application, all the stress values only indicate a sign reversal and are approximately the same in terms of magnitude.

#### 2.3.3. End Support

**Figure 14:**
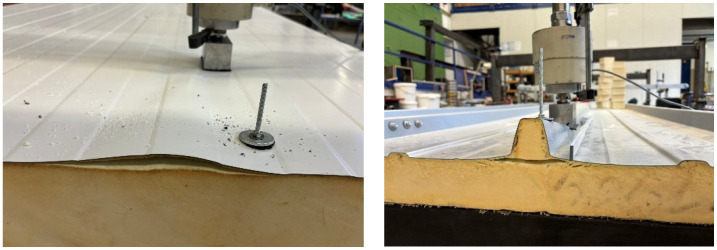
Failure at end support under compression: (**left**) PIR-L-140 10.0 kN; (**right**) PIR-T-60 5.5 kN.

When a load is applied near the end support of the two-span beam, a pull-off of the face or of the loaded rib, respectively, is observed (Figure 14).

As a result of the indentation caused by the point load in the core, lifting forces occur at the free end (negative moment in the rib). This effect does not occur at the mid-support, as the continuous face restricts the lift-off forces.

### 2.4. Cyclic Test

**Figure 15:**
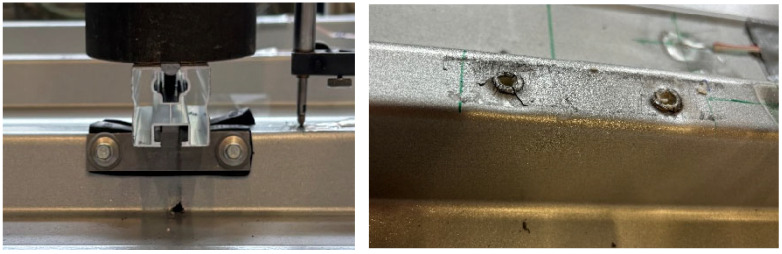
(**left**): Load introduction in cyclic tests; (**right**): Fatigue failure on upper flange of rib.

In order to simulate the alternating pressure–suction load from wind over the service life, a cyclic alternating load of ±1.5 kN was applied in a separate series of tests using an electronically controlled hydraulic cylinder. At least 10,000 cycles with a frequency of 1 Hz were applied. The tests were also carried out on 2 × 3 m two-span beams, where the load was always applied in the centre of the span (Figure 15 left).

After applying the load cycles, the load-bearing capacity under tension and subsequent compression was determined. A decrease in the resistance capacity under tension could generally not be observed. The measured values also remained within a constant corridor during the cyclic tests. Only the load application via a pair of bolts on the upper flange of the rib, which was also investigated, proved to be problematic from a fatigue point of view. In the two tests, the bolts pulled out under the halved top load of 0.75 kN, with clearly visible radial fatigue cracks around the bolt holes after around 1800 and 4500 cycles, respectively (Figure 15 right).

## 3. Numerical Studies

### 3.1. Modelling and Verification

**Figure 16:**
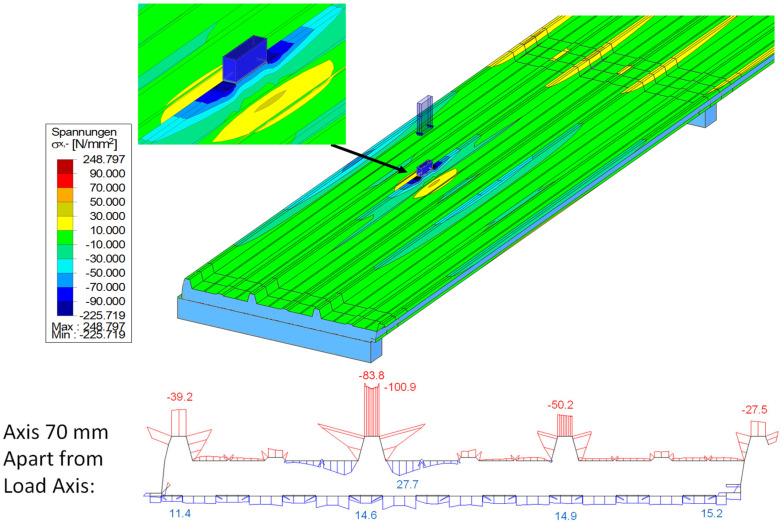
Resulting normal stresses in numerical model at 1.2 kN (PIR-T-60).

Normal stresses in the faces could already be deeply analysed due to the dense application of strain gauges. The shear stresses in the core and the faces, however, could not be measured during the tests. The determination of these shear stresses can only be performed numerically. Therefore, a numerical model [16] was developed using shell elements for the faces with their actual thicknesses, as well as volume elements for the core and supports (Figure 16).

Since the focus of the numerical investigations remained in the elastic range, the contact between the core and the faces is rigid, and the material properties of the steel and core materials are isotropic and linear elastic. The contact between the lower face and the supports provides only vertical support. Composite action is avoided by reducing the material properties of support volume to almost zero, with exception of E_Z_.

**Figure 17:**
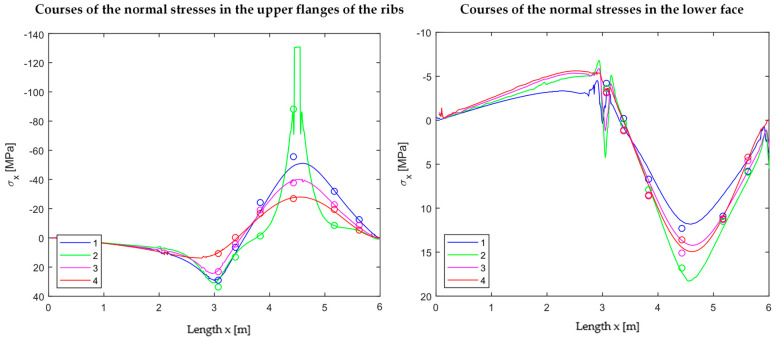
Normal forces in upper flanges of ribs (**left**) and lower face (**right**); PIR-T-60 at 1.2 kN.

The measured values could be used to validate the numerical model. As seen in Figure 16, the investigated bending behaviour with the tension effects at the bottom of the loaded rib can be reproduced. In Figure 17, the extracted normal stress curves in the upper face of the rib (left) and in the lower face (right) are compared to the corresponding discrete values (dots) from the tests.

The matching is sufficient. The relieving effect from the point load to the loaded rib in the neighbouring axes is also clearly shown in Figure 17 (left). In addition, the unloaded span shows no relevant stresses and can therefore be neglected for a superposition.

Since a huge amount of stress points is available in the validated numerical model, the stress resultants—face normal force and face bending moment—can be integrated over the entire width and compared to those calculated by a truss model (TM) or analytically.

The integrated stress resultants from bending, M_F_ and M_S_ = N_F_∙e, obtained from the numerical overall model (TotM) with 2 × 3 m span via stress integration over the entire face width, are compared with those from the truss model (load application over 100 mm line load). This comparison is displayed in the following figure for the central load position under the point load of 1.2 kN using the panel PIR-T-60.


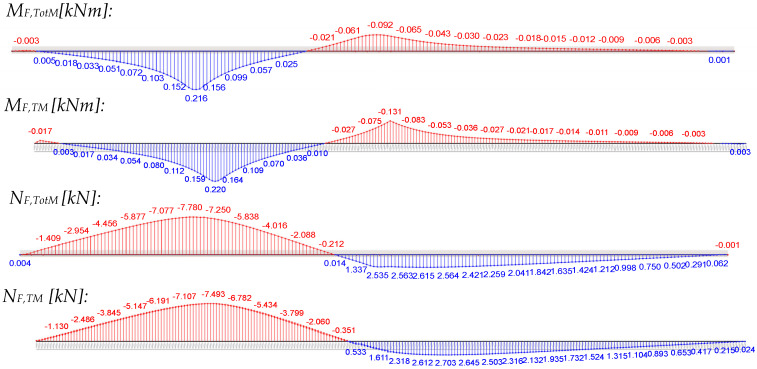


It can be seen that the courses can be reproduced both qualitatively and quantitatively by the truss model, which is much easier to model. Only the support moment is overestimated in the truss model due to the point support, as the favourable moment rounding because of the support width of 100 mm is not considered. This also leads to the conclusion that the integrated stress resultants from a concentrated point load are equal to those of a transversal line load, activating the entire cross-section without restriction. This finding is very important for the subsequent design approach, since its foundation relies on the use of the stress resultants from the panel with full width.

The numerical model can now be used to analyse the shear bearing behaviour of a sandwich panel subjected to a point load.

### 3.2. Shear Distribution

**Figure 18:**
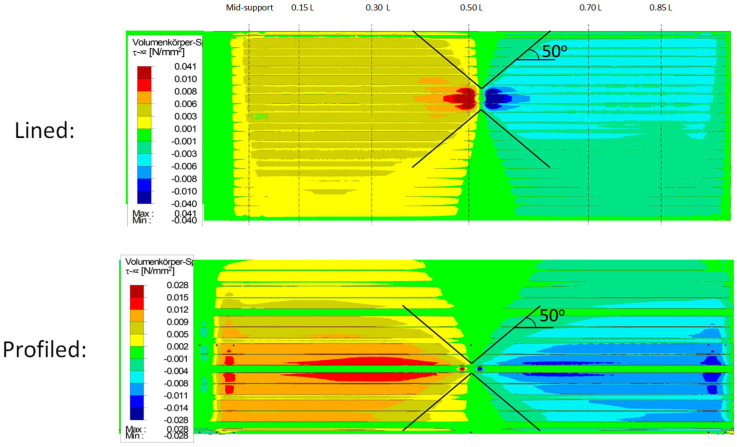
Qualitative shear stresses in core for flat and profiled panels.

In the case of wall panels without significantly profiled cover layers, the shear transfer is carried out solely via the core. In roof panels, the profiled surface layer takes over the majority of the shear transfer in the immediate area of the point load application. The shear stresses are then redistributed to the core as the longitudinal distance to the point load increases (Figure 2). The shear stresses in the core (Figure 18) show an approximately linear propagation at a 50° angle from the point load for both the wall and roof elements.

**Figure 19:**
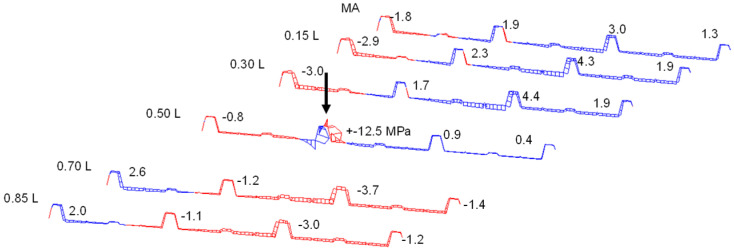
Shear stresses in profiled face (1.2 kN).

The shear stresses in the profiled face (Figure 19) confirm that all the shear is initially transferred via the webs of the directly loaded rib.

In the neighbouring sections adjacent to the point load, a low stress level in the face and a good stress distribution can already be determined due to the drop in the face shear force.

### 3.3. Parametric Study

The range of parameters is defined based on an overview of the sandwich panels currently on the market [17], which was published by the lightweight steel construction association, IFBS, with the technical approvals currently available from the DIBT. Geometric and material-specific properties vary based on the tested panels. In addition, the static system and the span are modified. The following overview shows the various parameters based on the properties of six test specimens:

- Cover sheet thicknesses: top: 0.6; 0.7 mm–bottom 0.5; 0.6 mm;
- Continuous Core thickness: 40, 60, 80, 100, 120, 140, 160 mm;
- Shear modulus and Modulus of elasticity: PIR: 2.5; 3.5; 4.5 MW: 7.3; 8.3; 9.3 MPa;
- Rib width (upper flange): 20, 25, 30 mm;
- Rib height: 32, 37, 42 mm;
- Number of ribs: 4, 5;
- Single-span beams: 3; 4; 5 m span;
- Two-span beams: 3; 4; 5 m span;
- Three-span girder: 3; 4; 5 m span;
- Cantilever: 0.5 m length.

The parameters are not combined here; they are only considered individually. The core moduli are analysed in pairs E_C_ = G_C_. The core thicknesses are compared with constant shear and modulus of elasticity.

The influence of each parameter is analysed on the basis of the effective widths at the centre load position. It is shown that the geometric properties of the faces (rib height and width, as well as face thickness) have no measurable influence on the effective widths. Similarly, the variation in the core shear modulus and the modulus of elasticity has no noticeable relevance to the effective widths.

The static system and the span, as well as the core thickness, have the greatest significance for the effective widths.

Another result of the parametric study is that the effective width of the face bending moment decreases with increasing core thickness (by 20% from d = 40 to 160 mm), and that of the face normal force increases slightly at the same time.

In addition, it can be said that the effective widths of the face bending moment rise slightly with increasing span width (approx. +10% from 3 to 5 m). This applies to all the static systems analysed. By adding a fifth rib, the effective widths for the face bending moment reduce by up to 20%.

## 4. Design Concept for Point Loads

**Figure 20:**
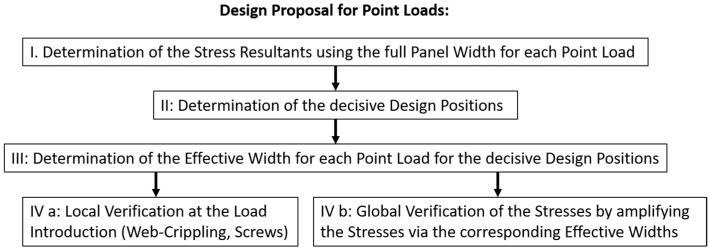
Design process for point loads (1.2 kN).

As shown by the failure modes, there should be a division between local and global failures. Figure 20 provides an overview of the design process.

For local failure under compression and tension, existing design concepts are used [18,19].

For compression, plastic crippling of the webs of the ribs was decisive for local failure. A local compression check for trapezoidal sheets showed realistic results for the sandwich panels in the tests.

*Local Compression (Web Crippling)*

Analogous to DIN EN 1993-1-3 [20] Chap. 6.1.7.3, we derive the following:

(2) $$R_{w,Rd}=\alpha \cdot t^{2}\cdot \sqrt{f_{yb}\cdot E}\cdot \left(1-0.1\cdot \sqrt{\frac{r}{t}}\right)\cdot \left[0.5+\sqrt{0.02\cdot \frac{l_{a}}{t}}\right]\cdot \frac{2.4+{\left(\frac{\phi }{90}\right)}^{2}}{\gamma_{M1}}$$

For tension, maximum load could be applied right before delamination at load introduction occurred. However, the calculated characteristic resistance of the screws based on the ETAs—considering the angle *ϕ* of the webs of the rib—was always below the load required for delamination. So, at this point, it was decided that the resistance of the screws would be taken as the decisive maximum tension load. Further investigations into the local tension effects are planned at the University of Wuppertal.

*Local Tension (Pull-Out of Screws)*


(3) $$\frac{F_{Ed}\cdot \cos\left(\phi \right)}{F_{T,Rd}}+\frac{F_{Ed}\cdot \sin\left(\phi \right)}{F_{V,Rd}}\leq 1.0\;$$

For global failure under compression, wrinkling has always been the decisive failure mode. A shear failure of the core was not witnessed in the tests, but it might occur for thinner wall panels when the loading is close to the support.

Based on the tests and the numerical parameter study, the following formulas for the effective widths for the normal force and the face bending moment, depending on the position x of the point load, were derived (Table 1). The effective widths for the core shear were chosen based on a 50° expansion from the point load. The shear in the profiled face was solely designated to the loaded rib, with linear spreading in the longitudinal direction.

These effective widths could be well integrated into the design concept of Eurocode 3—Part 7 [7].

## 5. Design Example

In order to demonstrate the application of the design approach, a two-span beam (2 × 3000 mm) with three point loads in one field will be considered as a practical example. The loads are introduced over a length of 100 mm. The other input arguments are as follows:

B = 1000 mm; d = 60 mm; e = 69 mm; G_c_ = 4.0 MPa; t_F,o_ = 0.56 mm; t_F,u_ = 0.46 mm; I_yy,tot,o_ = 14.98 cm^4^; W_y,min,tot,o_ = −4.49 cm^3^; A_tot,o_ = 7.13 cm^2^; A_tot,u_= 5.26 cm^2^; S_y,max,tot,o_ = 0.70 cm^3^, ϕ_web_ = 75°

First, local verification is performed at the load introduction (according to DIN EN 1993-1-3 [20] Equation 6.18).

$$R_{w,Rd}=2\cdot \left(0.15\cdot \frac{0.056^{2}\cdot \sqrt{32\cdot 21.000}}{1.1}\cdot \left(1-0.1\cdot \sqrt{7.1}\right)\cdot \left(0.5+\sqrt{\frac{0.02\cdot 10}{0.056}}\right)\cdot \left(2.4+{\left(\frac{75}{90}\right)}^{2}\right)\right)\;=3.8\;kN>1.2\;kN\;$$

Afterwards, the internal forces are calculated from all three individual loads (0.85 L; 0.50 L; 0.15 L) with 1.2 kN each.


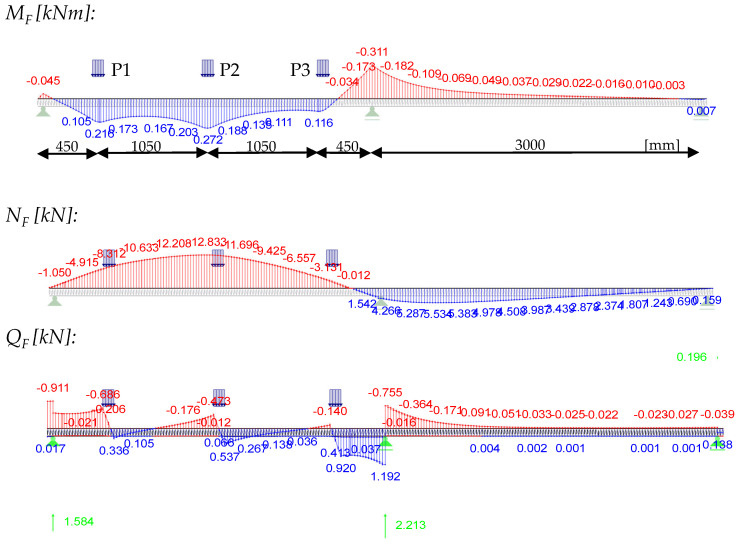


Determination of the decisive design points is not trivial due to stress propagation and the different effective widths of the point loads. The effective widths to be applied in the longitudinal distance to the point load are of decisive importance for the economic efficiency of the design method. The current draft standard recommends freezing the effective widths of a point load in the longitudinal direction. The stress pattern in the panel shows, however, that there is already an almost complete stress propagation in the face at a distance of more than 500 mm.

For this reason, a propagation is considered for the effective widths of the neighbouring point loads. The equation for the effective width at the centre bearing is used here, where the distance x corresponds to the distance of the point load to the verification point. This leads to the following effective widths and utilisation ratios:

Wrinkling in the field (at x = 0.5 L = 1500 mm):

For P2:

b_eff,MF,P2_ = (0.35 + 0.4·x/l)·B = (0.35 + 0.4·1500/3000)·1000 mm = 550 mm (p_1_ = p_2_ = 1.0)

b_eff,NF,P2_ = (0.4 + 0.6·x/l)·B =(0.4 + 0.6·1500/3000)·1000 mm = 700 mm

For P1 and P3:

b_eff,MF,P1_ = (0.3 + 0.6·(1050/3000))·1000 mm = 510 mm = b_eff,MF,P3 _ (p_1_ = p_2_ = 1.0)

b_eff,NF,P1_ = (0.4 + 0.6·(1050/3000))·1000 mm = 610 mm = b_eff,NF,P3_

This leads to the following stress components from the respective point loads, with their associated internal forces at the verification point in the centre of the span:

$$\sigma_{x,P2}=\frac{\frac{-7.5\;kN}{7.13\;cm^{2}}}{700\frac{mm}{1000}mm}+\frac{0.220\;kNm\cdot \frac{100}{-4.49\;cm^{3}}}{550\frac{mm}{1000}mm}=-10.4\frac{kN}{cm^{2}}=-104\;MPa$$

As the effective widths of P1 and P3 are identical for this design point, they are summarised.

$$\sigma_{x,Ed}=-138\;MPa<\sigma_{w,d}=-285\;MPa\;\left(\eta =48\;\%\right)\sigma_{x,P1+P3}\;\;=\frac{\frac{-3.0\;kN\;-\;2.2\;kN}{7.13\;cm^{2}}}{610\frac{mm}{1000\;}mm}+\frac{\left(0.03+0.02\right)\;kNm\cdot \frac{100}{-4.49\;cm^{3}}}{510\frac{mm}{1000\;mm}}\;=-34\;MPa=-3.4\frac{kN}{cm^{2}}$$

The check at midspan for wrinkling against the wrinkling strength can now be performed.

$$\sigma_{x,Ed}=-138\;MPa<\sigma_{w,d}=-285\;MPa\;\left(\eta =48\;\%\right)$$

Wrinkling at the mid-support (lower face):

b_eff,NF,P3_ = (0.4 + 0.6∙450/3000)∙1000 mm = 490 mm

b_eff,NF,P2_ = (0.4 + 0.6∙1500/3000)∙1000 mm = 700 mm

b_eff,NF,P1_ = (0.4 + 0.6∙2550/3000)∙1000 mm = 910 mm > 0.7∙B = 700 mm

$$\sigma_{x,P3}=\frac{\frac{-0.8\;kN}{5.26\;cm^{2}}}{490\frac{mm}{1000}mm}=-0.31\frac{kN}{cm^{2}}=-3.1\;MPa$$

$$\sigma_{x,P1+P2}=\frac{-\frac{2.1+1.0\;kN}{5.26\;cm^{2}}}{700\frac{mm}{1000}mm}=-0.84\frac{kN}{cm^{2}}=-8.4\;MPa$$

$$\sigma_{x,Ed}=-11.5\;MPa<\sigma_{w,d}=\frac{122}{1.12}=-109\;MPa\left(\eta =10\%\right)$$

Shear failure of the face at x = 0.15 L from mid support:

b_eff,QF,P1_ = 333 mm

b_eff,QF,P2,P3_ = 800 mm (already max. expansion)

$$\tau_{F,P2}=\frac{0.69\;kN\cdot 0.7\;cm^{3}}{14.9\;cm^{4}\cdot 0.056\;cm}\cdot \frac{1000\;mm}{333\;mm}=1.74\frac{kN}{cm^{2}}=17.4\;MPa$$

$$\tau_{F,P1+P3}=\frac{\left(0.18+0.06\right)\;kN\cdot 0.7\;cm^{3}}{14.9\;cm^{4}\cdot 0.056\;cm}\cdot \frac{1000\;mm}{800\;mm}=0.25\frac{kN}{cm^{2}}=2.5\;MPa$$

$$\tau_{F,Ed}=19.9\;MPa<\frac{320}{\sqrt{3}\cdot 1.1}=168\;MPa\left(\eta =12\%\right)$$

Shear failure of the core at mid support:

b_eff,QC,P1,P2,P3_ = 800 mm (already max. expansion)

$$\tau_{C,Ed}=\frac{2.21-0.19-0.88\;kN}{6\;cm\cdot 80\;cm}=0.0024\frac{kN}{cm^{2}}=0.024\;MPa<f_{Cv,d}=0.09\;MPa$$

$$\left(\eta =26\%\right)$$

Vertical compression at mid-support:

Vertical pressure verification is performed with the effective widths of the core shear.

$$\sigma_{C,z,Ed}=\frac{2.21\;kN}{10\cdot 0.8\cdot 100}=0.0028\frac{kN}{cm^{2}}=0.028\;MPa<f_{Cc,d}=\frac{0.10}{1.3}=0.077\;MPa\left(36\%\right)$$

## 6. Conclusions

The aim of this research project was to investigate the load-bearing behaviour of sandwich panels under point load application and to develop a purely calculative design concept for sandwich panels under point loads. In contrast to current design practice, which is based solely on the ultimate load condition, the focus here was on the elastic stress pattern under point loads in order to be able to make general manufacturer-independent statements and to enable a safe superposition with other load cases. The principle of effective widths was used to quantify the stress concentration in the sandwich cross-section.

In a series of tests on a two-span girder, with two lined and four profiled sandwich elements of different thicknesses and the core materials PIR and MW, the extensive application of strain gauges has provided precise information about the stress pattern in the surface layers. In the case of the lined panels, an indentation occurred under the point load even at a low load level, which strongly influenced the load-bearing behaviour in the upper face (membrane).

In the ultimate load tests, the decisive mode for failure under compression was always the wrinkling of the upper face. Under tension, local delamination initially occurred at the load introduction. The total failure was then the screw pull-out.

In the cyclic tests, even after 10,000 load cycles (compression/tension) at the service load level, no significant redistribution or load-reducing effects were detected. Only the connection on the top flange of the rib proved to be unsafe under cyclic loading, as the failure mode of bolt pull-out perpendicular to the cover plate had extremely low fatigue strength.

In order to be able to make statements about the shear stresses in the profiled face and in the core, the tests carried out were modelled numerically. The propagation of the shear in the core was almost linear from the point load. For the roof panels, a distinction must also be made between the shear force of the surface layer and the core. In the area of the point load, the shear force was transferred solely via the profiled face, as in the analytical solution. In addition to that, only the loaded rib was activated by the local load application. In the longitudinal direction, the shear force bearing components then shifted towards the core component, and the shear stresses propagated in the face and core.

One additional important finding of the numerical model was that the total internal forces on the sandwich panel corresponded to those of a line load across the entire width, and that there was no redistribution of the sandwich/profiled face proportions in the roof panels. The total internal forces could therefore be determined using the truss model, for example. The stress maxima due to the local load application could then be determined by increasing the stresses with the effective widths obtained for each partial section size.

The stress pattern under point tension differed only in the sign of the result variables for the compressive stress, which makes the design much easier.

Finally, a purely calculative design concept was presented on the basis of the effective widths, which considered the stress concentration on the action side and utilised the existing resistances from the sandwich approvals determined for the full panel-width on the resistance side. The internal forces could initially be determined for each individual load on the truss model. The stresses were then determined using the total cross-section values, increased for each partial stress resultant with the corresponding effective width and compared with the permissible limit stresses. For local verification, the existing design concepts against web crippling of the rib (EN 1993-1-3 for trapezoidal profiles) and the respective ETAs for the screw connections were used.

This allowed a purely mathematical design for point-loaded sandwich panels, with cross-manufacturer applicability in the elastic range. The design concept aligns well with the design process of the new Eurocode for sandwich panels. Significantly higher load-bearing resistances can still be determined using ultimate load tests, which can be problematic in the case of old existing roofs. However, these high resistances rarely have to be utilised due to the often closely arranged load application points in practice and thus relatively low individual load values.

A more detailed presentation of the research results can be found in [9].

The local delamination at the load introduction under tensile loads, which could not yet be quantified, is the subject of further investigations at the University of Wuppertal.

## Figures and Tables

**Table 1:** Effective widths.

**One Span**						
Case:	Q_F_	Q_C_		M_F_		N_F_
Support	e_R_ + x ≤ 0.8 B	b_e_ + d_C_ + 1.2∙x ≤ 0.8 B		-		-
In Span	e_R_	b_e_ + d_c_		(0.4 + 0.2∙x/L)∙B		(0.5 + 0.65∙x/L)∙B
**Multi Span—End-Field**						
Case:	Q_F_	Q_C_		M_F_		N_F_
Support	e_R_ + x ≤ 0.8 B	b_e_ + d_C_ + 1.2∙x ≤ 0.8 B		(0.3 + 0.6∙x/L)∙B ≤ 0.7 B		(0.4 + 0.6∙x/L)∙B ≤ 0.7 B
In Span	e_R_	b_e_ + d_c_		(0.35 + 0.4∙x/L)∙B		(0.4 + 0.6∙x/L)∙B
**Multi Span—Inner-Field**						
Case:	Q_F_	Q_C_		M_F_		N_F_
Support	e_R_ + x ≤ 0.8 B	b_e_ + d_C_ + 1.2∙x ≤ 0.8 B		(0.35 + 0.7∙x/L)∙B ≤ 0.7 B		(0.45 + 0.65∙x/L)∙B ≤ 0.7 B
In Span	e_R_	b_e_ + d_C_		(0.35 + 0.3∙x/L)∙B		(0.45 + 0.65∙x/L)∙B
**Cantilever**						
Case:	Q_F_	Q_C_		M_F_		N_F_
Support	e_R_ + x ≤ 0.8 B	b_e_ + d_C_ + 1.2∙x ≤ 0.8 B		0.35 + 0.2∙x ≤ 0.7 B		0.45 + 0.4∙x ≤ 0.8 B
In Span	e_R_	b_e_ + d_C_		-		-
**Notes:** e_R_ = Transversal distance of ribs b_e_ = Application width of point load, width of rib on bottom for profiled panels, respectively d_C_ = core thickness L = span, in which the point load is applied The effective widths of the face bending moment must then be multiplied by the coefficients p_1_ and p_2_, depending on the core thickness and span						
Core Thickness [mm]		p_1_	Span in the Loaded Field [m]		p_2_	
<80		1.0	≤3		1.0	
80–120		0.9	4		1.05	
>120		0.8	≥5		1.1	
For point loads applied at the edge of the panel, the effective widths must be halved. For profiled panels with five ribs, the effective widths of the face bending moment must be multiplied by 0.8. For the superposition of several point loads, the distance x of the point load to the considered section may be used for all point loads outside the considered section in the field, and the equation of the effective width for supports may be used.						

## Data Availability

The data presented in this study are available on request from the corresponding author.

## Glossary

*A*	area
*B*	full panel width
*b_e_*	application width of the point load, width of the rib on the bottom for profiled panels respectively
*b_eff_*	effective widths
*d_C_*	core thickness
*e*	distance between the centroids of the faces
*e_R_*	transversal distance of the ribs
*L*	span, in which the point load is applied
*M_F_*	bending moment of the face
*N_F_*	normal force of the face
t_F,o_	design thickness of the upper face
t_F,u_	design thickness of the lower face
Q_C_	shear force of the core
Q_F_	shear force of the profiled face
ϕ_web_	angle of the web of the rib
